# Decreased levels of the gelsolin plasma isoform in patients with rheumatoid arthritis

**DOI:** 10.1186/ar2520

**Published:** 2008-09-27

**Authors:** Teresia M Osborn, Margareta Verdrengh, Thomas P Stossel, Andrej Tarkowski, Maria Bokarewa

**Affiliations:** 1 Department of Rheumatology and Inflammation Research, University of Gothenburg, Guldhedsgatan 10A, S-413 46 Gothenburg, Sweden; 2 Translational Medicine Division, Brigham and Women's Hospital, Department of Medicine, Harvard Medical School, 75 Francis Street, Boston, MA 02115, USA

## Abstract

**Introduction:**

Gelsolin is an intracellular actin-binding protein involved in cell shape changes, cell motility, and apoptosis. An extracellular gelsolin isoform, plasma gelsolin circulates in the blood of healthy individuals at a concentration of 200 ± 50 mg/L and has been suggested to be a key component of an extracellular actin-scavenging system during tissue damage. Levels of plasma gelsolin decrease during acute injury and inflammation, and administration of recombinant plasma gelsolin to animals improves outcomes following sepsis or burn injuries. In the present study, we investigated plasma gelsolin in patients with rheumatoid arthritis.

**Methods:**

Circulating and intra-articular levels of plasma gelsolin were measured in 78 patients with rheumatoid arthritis using a functional (pyrene-actin nucleation) assay and compared with 62 age- and gender-matched healthy controls.

**Results:**

Circulating plasma gelsolin levels were significantly lower in patients with rheumatoid arthritis compared with healthy controls (141 ± 32 versus 196 ± 40 mg/L, *P *= 0.0002). The patients' intra-articular plasma gelsolin levels were significantly lower than in the paired plasma samples (94 ± 24 versus 141 ± 32 mg/L, *P *= 0.0001). Actin was detected in the synovial fluids of all but four of the patients, and immunoprecipitation experiments identified gelsolin-actin complexes.

**Conclusions:**

The plasma isoform of gelsolin is decreased in the plasma of patients with rheumatoid arthritis compared with healthy controls. The reduced plasma concentrations in combination with the presence of actin and gelsolin-actin complexes in synovial fluids suggest a local consumption of this potentially anti-inflammatory protein in the inflamed joint.

## Introduction

Plasma gelsolin (pGSN) is the extracellular isoform of a ubiquitous cytoplasmic actin-binding protein, gelsolin (GSN), that mediates cell shape changes and motility [1]. Differentially processed mRNA transcripts present in various cell types [2,3] and originating from a gene on chromosome 9 program the synthesis of intracellular gelsolin (cGSN) and of its secreted counterpart. The two isoforms are structurally and functionally identical except for 25 additional amino acids at the N terminus of pGSN [4]. pGSN circulates in the plasma of healthy humans and other mammals at average levels of 200 ± 50 mg/L. In every acute tissue injury setting examined, including toxic, hyperoxic, and idiopathic lung injury, adult respiratory distress syndrome, acute liver injury, myonecrosis, pancreatitis, trauma, burns, and bacterial and protozoal sepsis, pGSN levels are subnormal [5-14].

The unifying explanation for low pGSN concentrations in acute inflammatory conditions is the exposure by injury to plasma of the GSN-binding ligand, actin, a major cellular constituent ordinarily separated from the extracellular environment by intact plasma membranes. In some but not all such cases of pGSN depletion, GSN-actin complexes are detectable in the circulation. pGSN together with Gc-globulin, another extracellular actin-binding protein, is proposed to function as an 'extracellular actin scavenger system' responsible for the removal of actin released during tissue injury [15]. Actin exposed to the extracellular environment polymerizes into filaments (F-actin) that stimulate downstream inflammatory reactions [16]. pGSN has the capacity to sever and depolymerize F-actin into monomeric subunits (G-actin) that are then sequestered by Gc-globulin [17] and cleared in the liver [18,19]. Administration of pGSN to animals subjected to systemic inflammation can prolong survival and prevent complications of acute injury [12,14,20]. The beneficial effect of pGSN in these settings is unclear but may reside in its binding and/or inactivation of inflammatory mediators such as lysophosphatidic acid, amyloid β protein, diadenosine 5',5"'-P1,P3-triphosphate, endotoxin, and platelet-activating factor) [21-26]. These findings suggest that pGSN is a broad-spectrum anti-inflammatory buffer and that local pGSN depletion by a shift of binding toward actin during actin exposure following injury allows mediators to promote appropriate defense and repair functions. Catastrophic or prolonged pGSN depletion, however, hypothetically accommodates dysfunctional and destructive actions of the mediators, leading to secondary organ damage and even death.

This set of events is theoretically also applicable to chronic inflammatory conditions in which cellular damage and mediator release occur, but no studies have hitherto examined pGSN levels in such states. Rheumatoid arthritis (RA) is a chronic autoimmune disease of unknown etiology that most prominently affects the synovial lining, resulting in a persistent and progressive diarthrodial joint inflammation and destruction. We report here that pGSN levels are diminished in the blood of RA patients and that analysis of synovial fluids (SFs) suggests that pGSN is consumed in the inflamed joint. Our findings suggest that the reason for the decreased pGSN levels is local exposure of actin to the extracellular environment in these joints.

## Materials and methods

### Patients

Plasma and SF samples were collected from RA patients attending the rheumatology clinics at Sahlgrenska University Hospital in Gothenburg for acute joint effusion. Altogether, samples were obtained from 81 patients. Three of the patients donated SF only. RA was diagnosed according to the American College of Rheumatology criteria [27]. Clinical and demographic data of the RA patient population are presented in Table 1. At the time of SF and blood sampling, all of the patients received non-steroidal anti-inflammatory drugs. Disease-modifying anti-rheumatic drugs (DMARDs) were administered to 45 patients, including methotrexate (MTX) (33 patients), sulfasalazine (5 patients), leflunomide (1 patient), parenteral or oral gold salt compounds (4 patients), cyclosporine A (5 patients: 2 in combination with MTX, 1 in combination with leflunomide, 1 in combination with azathioprine, and 1 with sulfasalazine), and azathioprine (2 patients). Nine patients received a combination of DMARD (8 patients received MTX and 1 patient received azathioprine + cyclosporine A) and inhibitors of tumor necrosis factor-alpha (5 patients received infliximab and 4 patients etanercept). One patient received MTX in combination with a soluble interleukin-1 (IL-1) receptor agonist (anakinra). The remaining 33 patients had no DMARD treatment at the time of blood and SF sampling. Thirty-one patients used oral glucocorticosteroids (mean dose of 6.85 mg/day). Patients receiving monotherapy with glucocorticosteroids (n = 12) were considered as having no DMARD treatment. Recent radiographs of the hands and feet were obtained for all patients. The presence of bone erosions defined as the loss of cortical definition of the proximal interphalangeal, metacarpophalangeal, carpal, interphalangeal, and metatarsophalangeal joints was documented: a single erosion was defined as erosive disease. The presence of rheumatoid factor of any of the immunoglobulin isotypes tested (IgM, IgA, and IgG) was considered as positive. The study was approved by the Ethical Committee of the University of Gothenburg. Informed consent was obtained from all patients and volunteers enrolled in this study in accordance with the Declaration of Helsinki. Control blood samples (n = 62) were obtained from volunteers donating blood at the Blood Transfusion Unit of Sahlgrenska University Hospital and matching the RA patients for age and gender.

**Table 1:** Clinical characteristics of patients with rheumatoid arthritis

Clinical characteristic	Erosive RA (n = 47)	Non-erosive RA (n = 31)	*P *value^a^
Gender, female/male	35/12	24/7	NS

Age, years	61.2 ± 14.1	54.2 ± 7.4	NS

Rheumatoid factor, +/-	35/12	12/19	0.008

Disease duration, years	12.1 ± 9.2	7.7 ± 7.4	0.005

Treated with DMARDs	32	13	<0.03
Methotrexate	24 (51%)	9 (29%)	
Other^b^	8 (17%)	4 (13%)	
Non-treated	15 (32%)	18 (58%)	<0.05

CRP, mg/L	42 ± 56.6	38 ± 39.6	NS
Systemic inflammation, CRP >20 mg/L	28 (60%)	18 (58%)	

White blood cell count, × 10^9^/L			
Blood	8.2 ± 2.8	7.34 ± 1.1	NS
Synovial fluid	10.6 ± 17.7	13.0 ± 14.1	NS

Values are mean ± standard deviation except where otherwise indicated. ^a^Erosive versus non-erosive. ^b^Sulfasalazine 5, gold salts 4, leflunomide 1, cyclosporine A 5 (in combination with methotrexate 2, with azathioprine 1, with leflunomide 1, and with sulfasalazine 1), and azathioprine 2. CRP, C-reactive protein; DMARD, disease-modifying anti-rheumatic drug; NS, not significant; RA, rheumatoid arthritis.

### Collection and preparation of samples

SF was obtained from knee joints by arthrocentesis, aseptically aspirated, and transferred into tubes containing sodium citrate (0.129 mol/L, pH 7.4). Blood samples were simultaneously obtained from the antecubital vein and collected into sodium citrate anti-coagulant. Blood and SF samples were centrifuged at 800 *g *for 15 minutes. Supernatants were collected, separated into lots, and stored frozen at -70°C until use.

### Measurements of plasma gelsolin concentrations in plasma and synovial fluid

pGSN was quantified functionally by its ability to promote the nucleation of actin filament assembly using a fluorometric assay as previously described [14]. The assay is based on the principle that calcium-activated pGSN binds pyrene-labeled actin monomers to form a nucleus from which actin polymerizes in the pointed (slowest-growing) end direction. Pyrene-labeled actin fluoresces with higher intensity as a polymer than as a monomer. Pyrene actin was prepared by derivatizing actin with *N*-pyrenyliodoacetamide (Molecular Probes, now part of Invitrogen Corporation, Carlsbad, CA, USA) using the procedure of Kouyama and Mihashi [28], exchanging CaCl_2 _for MgCl_2_. Before use, pyrene actin was diluted in depolymerization buffer (buffer A: 0.5 mM ATP, 0.5 mM β-mercaptoethanol, 2 mM Tris, 0.2 mM CaCl_2_, pH 7.4) to 20 μM, stored 1 hour at 37°C to reach monomer equilibrium, and centrifuged at 250,000 *g *and 4°C for 30 minutes in an Optima™ TL Ultracentrifuge (Beckman Coulter, Inc., Fullerton, CA, USA) to pellet any remnant F-actin. The supernatant was withdrawn and stored in an ice water bath until use. Plasma or SF to be analyzed was diluted 1:5 in polymerization buffer (buffer B: 0.1 M KCl, 0.2 mM MgCl_2_, 1.5 mM CaCl_2_, 0.5 mM ATP, 10 mM Tris, 0.5 mM β-mercaptoethanol, pH 7.4). Pyrene-actin fluorescence was recorded using a spectrofluorometer (FluoroMax-2^®^; JobinYvon-Spex Instruments S.A., Inc, now HORIBA Jobin Yvon Inc, Edison, NJ, USA). Excitation and emission wavelengths were 366 and 386 nm, respectively. Pyrene actin was added to a final concentration of 1 μM in 280 μL of buffer B containing 0.4 μM phallacidin and 5 μL of diluted sample in 6 × 50 mm borosilicate glass culture tubes (Kimble, Glass Inc, Vineland, NJ, USA). Nucleation was monitored for 240 seconds in the fluorometer following a fast vortex. The linear slope of the fluorescence increase was calculated between 100 and 200 seconds. All of the samples were run in duplicates. Polymerization rate in each sample was converted to pGSN concentration by use of a standard curve of recombinant human pGSN (rhpGSN).

### Measurements of interleukin-6 levels in synovial fluid

The levels of IL-6 in SF were determined by a bioassay with a cell clone B13.29, subclone B9, which is dependent on IL-6 for growth, as described previously [29]. The samples were tested in 250-fold dilutions and compared with a standard curve obtained using human recombinant IL-6 (Genzyme, Kent, UK).

### Measurements of albumin in plasma and synovial fluid

Albumin was measured by use of a kit (QuantiChrom™ BCG Albumin Assay Kit; BioAssay Systems, Hayward, CA, USA) according to the manufacturer's instructions.

### Immunoblotting for gelsolin isoform in synovial fluid and gelsolin fragments in plasma

Platelet-poor plasmas or SFs were diluted 1:100 in 1× sample buffer (SB) (10% glycerol, 2% SDS, 62.5 mM Tris-HCl, 0.03% Bromphenol blue, 5% β-mercaptoethanol, pH 6.8) to detect GSN isoforms and 1:40 to document pGSN fragments, vortexed briefly, and incubated at 97°C for 5 minutes. Samples (10 μL for GSN isoform and 20 μL for GSN fragments) were run on 10% SDS-PAGE gels in a modified Laemmli system [30] and transferred to Immobilon P membranes (polyvinylidene difluoride [PVDF]) (0.45 μm) (Millipore Corporation, Billerica, MA, USA). Platelet lysate (2 × 10^8^/mL, 5 μL) and rhpGSN served as negative and positive controls for pGSN, respectively. For determination of GSN isoform, a polyclonal antibody recognizing an epitope in the plasma extension of human pGSN was used (1:2,000, 2 hours, 22°C). The antibody was designed using a peptide from the plasma extension sequence and produced by Invitrogen Corporation in rabbit using a KLH (keyhole limpet hemocyanin) carrier. The antibody titer was checked at 4, 8, and 10 weeks, and the antibody was affinity-purified. For total GSN, the mouse monoclonal anti-GSN antibody 2c4 was used [31,32] (1:2,500, 2 hours, 22°C). Secondary antibodies used were goat anti-rabbit IgG (H+L)-horseradish peroxidase (HRP) (1:5,000, 80 minutes, 22°C) and goat anti-mouse IgG (H+L)-HRP conjugate, respectively (1:3,300, 80 minutes, 22°C) (Bio-Rad Laboratories, Inc., Hercules, CA, USA). Chemiluminescence detection was done using SuperSignal^® ^West Pico Chemiluminescent Substrate for detection of HRP (Pierce, Rockford, IL, USA). HyBlot CL autoradiography film (Denville Scientific Inc., Metuchen, NJ, USA) was exposed to the membrane for 1 minute (isoform detection) or overnight (matrix metalloproteinase [MMP]-cleavage product detection). The film was developed using an M35A X-OMAT Processor (Eastman Kodak Company, Rochester, NY, USA).

The 2c4 antibody recognizes the C-terminal half of the GSN molecule and was used for detection of approximately 42- to 46-kDa fragments by immunoblotting as previously reported [33]. To confirm that the 2c4 antibody recognizes pGSN cleaved by MMPs into fragments and that cleavage can occur in plasma, rhpGSN (115 nM) or dilute human plasma (approximately 115 nM pGSN) was incubated with catalytic domain of MMP-3 (cMMP-3) (230 nM) (Sigma-Aldrich, St. Louis, MO, USA) in 50 mM Tris-HCl, 150 mM NaCl, 5 mM CaCl_2_, and 0.5 mM ZnCl_2_, pH 7.5, for various time points. SDS-PAGE and Western blots were performed as described above.

### Identification of actin in synovial fluid and plasma

SFs and plasmas were pre-cleared by centrifugation at 2,500 *g *for 5 minutes, diluted 1:20 and 1:10 in 1 × SB, respectively, and boiled for 10 minutes at 97°C. Samples (20 μL) were analyzed by 12% SDS-PAGE and transferred to PVDF membranes as described above. Actin was identified using a primary mouse monoclonal anti-β-actin antibody (Clone AC-15 Mouse Ascites Fluid, 1:1,000, 2 hours, 22°C) (Sigma-Aldrich) and a secondary (H+L)-HRP conjugated goat anti-mouse IgG (1:3,300, 80 minutes, 22°C) (Bio-Rad Laboratories, Inc.). Chemiluminescence detection was performed as described above, exposing the film to the membrane for less than 5 minutes. Quantification of actin in SFs and plasma was performed by densitometry using a human actin standard (actin protein, non-muscle) (Cytoskeleton, Inc., Denver, CO, USA) and Scion Image 1.62a software (Scion Corporation, Frederick, MD, USA).

### Detection of gelsolin-actin complexes in synovial fluid by immunoprecipitation

Eleven SFs that were strongly positive for actin were centrifuged at 2,500 *g *to pellet any cellular debris. Fifty microliters of supernatant was withdrawn and diluted 1:8 in binding buffer (20 mM Tris, 100 mM NaCl, 1 mM CaCl_2_, 0.01% Tween 20, pH 7.4). Samples were pre-cleared by incubation with 20 μL of GammaBind Plus Sepharose (GE Healthcare, Little Chalfont, Buckinghamshire, UK) 50/50 bead slurry for 1 hour end-over-end at 4°C to minimize unspecific interactions with the beads. After centrifugation to pellet beads, pre-cleared supernatants were removed and incubated end-over-end for 1 hour at 22°C with affinity-purified mouse anti-GSN IgG (2c4, 5 μg/mL). An unspecific mouse IgG was used as a control (normal mouse control IgG, sc-2025) (Santa Cruz Biotechnology, Inc., Santa Cruz, CA, USA) (5 μg/mL). Twenty microliters 50/50 bead slurry was added and incubation continued for 1 hour end-over-end. Beads were pelleted and washed four times in binding buffer before being resuspended in 50 μL of 1 × SB, boiled for 10 minutes, and analyzed by SDS-PAGE as described in the section above. Actin was identified using a rabbit polyclonal IgG (anti-actin N-terminal antibody produced in rabbit 1:750, 12 hours, 4°C) (Sigma-Aldrich) and a secondary (H+L)-HRP conjugated goat anti-rabbit IgG (1:2,000, 80 minutes, 22°C) (Bio-Rad Laboratories, Inc.). Chemiluminescence detection was performed as described above, exposing the film to the membrane for 2 minutes.

### Statistics

The levels of pGSN in the blood and SF samples were expressed as mean ± standard deviation and as median with interquartile range. Comparisons between the matched blood and SF samples were analyzed by paired *t *test. Comparison of pGSN and albumin levels was also performed between the patient blood samples and the healthy controls. For further comparison, patient material was stratified according to radiological findings (erosive RA versus non-erosive RA). For the evaluation of a possible influence of therapeutic interventions on pGSN levels, patients were stratified according to DMARD treatment (treated versus untreated). Differences in pGSN levels in the blood and SF between the groups were calculated separately employing the Mann-Whitney *U *test. Spearman correlation was used to determine the association between pGSN and albumin levels in blood and SF (GraphPad Prism software; GraphPad Software, Inc., San Diego, CA, USA). For all of the statistical evaluation of the results, *P *values of below 0.05 were considered statistically significant.

## Results

### Plasma gelsolin levels are significantly lower in plasma of patients with rheumatoid arthritis compared with matched healthy controls

**Figure 1:**
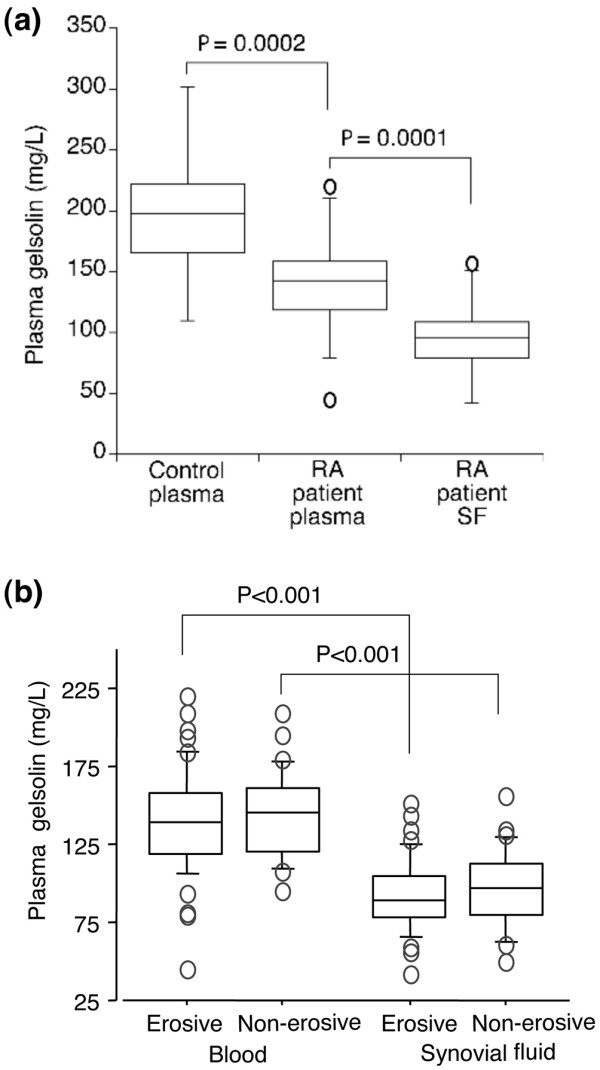
Plasma gelsolin concentrations in plasma and synovial fluids (SFs). **(a) **Plasma from matched rheumatoid arthritis (RA) patients (n = 78) and controls (n = 62) as well as SFs from RA patients (n = 81) were analyzed for plasma gelsolin concentration by a pyrene-actin nucleation assay. Data are shown as median (line in box: 197.3 mg/L for healthy controls, 142.8 mg/L for patient blood, and 95.6 mg/L for patient SF) and interquartile range (borders of box). Open dots denote outliers. Samples were run in duplicates. **(b) **Patient data stratified according to erosive and non-erosive RA.

Circulating pGSN levels were significantly diminished in patients with RA compared with those of matched healthy controls (141 ± 32 versus 196 ± 40 mg/L, *P *= 0.0002) (Figure 1a). A comparison of circulating pGSN levels between RA patients with erosive (pGSN plasma: 139.9 ± 34.1 mg/L, pGSN SF: 92.1 ± 23.9 mg/L) and non-erosive (pGSN plasma: 143.2 ± 28.2 mg/L, pGSN SF: 96.5 ± 24.4 mg/L) joint disease revealed no significant differences (Figure 1b). pGSN levels were similar in both males and females and were not dependent on the age of the patients or on the duration of arthritis. We observed no correlation between pGSN level and anti-rheumatic treatment. Circulating pGSN levels were inversely correlated to the levels of C-reactive protein (CRP) (*r *= -0.272, *P *= 0.026) but not related to other markers of inflammation investigated, including white blood cell counts and serum amyloid A protein (data not shown). In accordance with other studies [34], plasma albumin concentrations were lower in patients with RA compared with the controls (4.2 ± 0.6 versus 5.9 ± 0.6 g/dL, *P *< 0.0001) and lower in patients' SF than in plasma (2.2 ± 0.5 versus 4.2 ± 0.6 g/dL, *P *< 0.0001). pGSN levels in plasma and patient SF correlated positively with albumin levels (*r *= 0.46, *P *< 0.0001 in patient plasma, *r *= 0.35, *P *= 0.0051 in control plasma, and *r *= 0.2726, *P *= 0.0144 in SF).

To assess the impact of systemic inflammation on circulating pGSN levels, patients were stratified according to CRP levels, where CRP of above 20 mg/L indicated the presence of systemic inflammation. The pGSN levels had a tendency to be lower in circulation (135.8 ± 32.2 versus 148.9 ± 29.9 mg/L, *P *= not significant) and in SF (92.2 ± 24.4 versus 96.3 ± 23.7 mg/L, *P *= not significant) of patients having systemic inflammation as compared with those without. To evaluate a relationship between intra-articular levels of pGSN and local inflammation, we measured levels of IL-6 in SFs. The mean IL-6 level was 1.59 ± 0.11 ng/mL (range of 0.03 to 4.52 ng/mL), indicating some degree of local inflammation in most of the samples. However, pGSN levels showed no correlation with IL-6 levels.

### Characteristics of gelsolin present in plasma and synovial fluid of patients with rheumatoid arthritis

**Figure 2:**
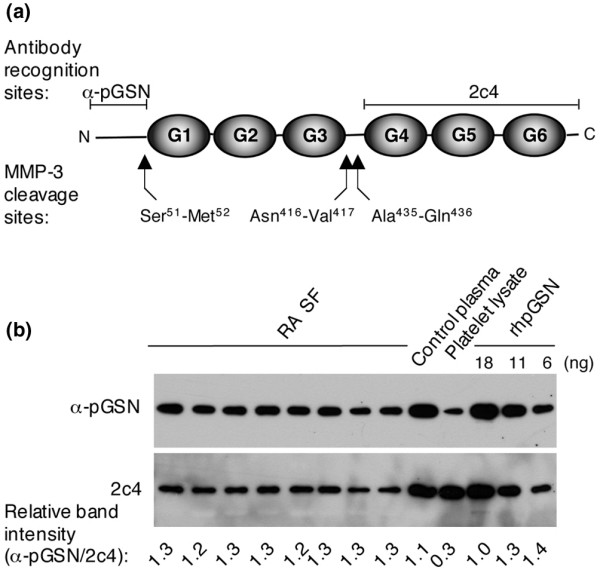
Determination of gelsolin isoform in synovial fluid (SF) by immunoblotting. **(a) **Schematic of the six-domain plasma gelsolin (pGSN) molecule. The respective epitopes for antibodies used are indicated. The 2c4 antibody recognizes the C-terminal half of the GSN molecule and thus both cytoplasmic gelsolin (cGSN) and pGSN. The α-pGSN antibody recognizes the N-terminal plasma extension specific for pGSN. Matrix metalloproteinase-3 (MMP-3) cleavage sites [33] are also indicated. **(b) **Identical samples were run on two separate gels and probed with rabbit IgG recognizing only the plasma isoform or the 2c4 antibody that recognizes both cytoplasmic and plasma isoforms. Band density was measured by Scion Image 1.62a software, and densities obtained for the two antibodies were compared. Data from representative immunoblots are shown. The relative band intensity is higher for the α-pGSN antibody for all of the SF samples and the plasma sample but much lower for the platelet lysate, which contains mainly the cGSN isoform. This indicates the specificity of the α-pGSN antibody for the pGSN isoform and shows that the main isoform in SF is pGSN (n = 3; 24 SF samples were analyzed). RA, rheumatoid arthritis; rhpGSN, recombinant human plasma gelsolin.

Immunoblots of GSN in SF of RA patients indicated that the GSN present in SF consists predominantly of the plasma isoform (Figure 2). Plasma origin of GSN was also supported by a correlation between GSN levels in plasma and SF in the matched pair of samples (*r *= 0.39, *P *= 0.0006). However, pGSN functional activity in SF from RA patients was significantly lower than that of plasma (94 ± 24 versus 141 ± 32 mg/L, *P *= 0.0001) as detected by its ability to promote actin filament assembly.

**Figure 3:**
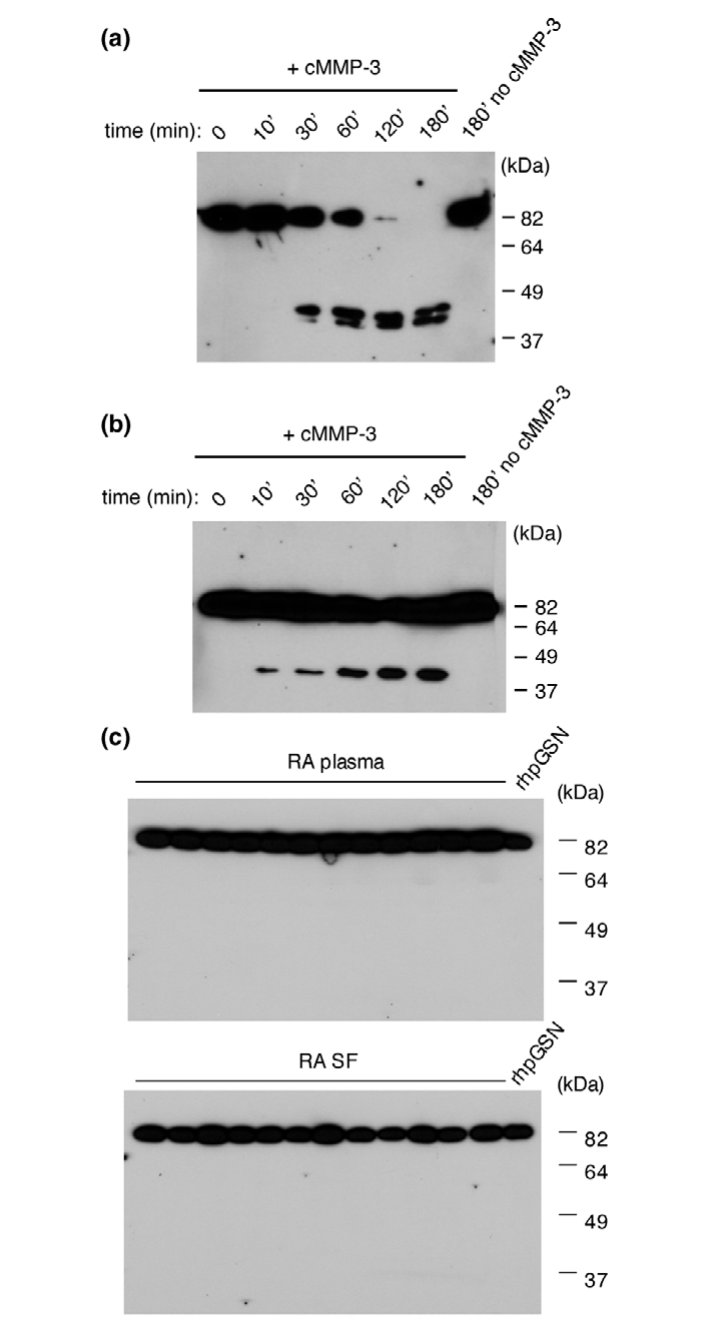
Immunoblots of plasma gelsolin (pGSN) cleavage products generated by matrix metalloproteinase (MMP).**(a) **The catalytic domain of MMP-3 (cMMP-3) was incubated with recombinant human pGSN (rhpGSN) at a 2:1 cMMP-3/pGSN ratio for various time points at 37°C. pGSN is cleaved and cleavage products of approximately 42 to 46 kDa are detected after 30 minutes of incubation using the 2c4 antibody, which recognizes the C-terminal half of GSN (Figure 2a) (n = 3). **(b) **cMMP-3 was incubated with human plasma from healthy controls for various time points at 37°C. More GSN cleavage products are visible with increasing incubation time, indicating cleavage and showing that cleavage products generated by MMP-3 should be detected in plasma if present at high enough quantities. (Note that hrpGSN or plasma incubated without cMMP-3 for 180 minutes does not show breakdown products.) The amount of pGSN added in (a) and (b) corresponds approximately to the average amount of pGSN added in (c). **(c)** Immunoblots of plasma and synovial fluid (SF) from randomly selected rheumatoid arthritis (RA) patient samples. MMP cleavage products are approximately 42 to 46 kDa in size and are recognized by 2c4 IgG (a, b) [33]. No cleavage products were observed in the patient samples (36 different plasma and SF samples were tested in three independent experiments). Representative immunoblots are shown.

*In vitro*, pGSN can be cleaved by MMPs into fragments of 42 to 46 kDa (at Asn^416^-Val^417^, Ser^51^-Met^52^, and Ala^435^-Gln^436^) [33,35]. In RA, MMPs (primarily MMP-1 and MMP-3) are elevated in both synovial tissue and serum [36]. Human recombinant pGSN as well as pGSN in plasma is cleaved *in vitro *upon addition of cMMP-3 [33]. Fragments are detected by the 2c4 antibody [33] (Figure 3a,b) and thus should be recognized if present in plasma or SF from RA patients. The same total amount of pGSN was added to wells in the gels showing *in vitro *cleavage as is present in gels showing the plasma and SF samples, and thus if cleavage products were present in the patient samples to a large extent, they would be detected. However, the evaluation of plasma and SF for evidence of proteolytic fragments of pGSN revealed no cleavage products of pGSN detectable by SDS-PAGE after immunoblotting (Figure 3c).

### Actin and gelsolin-actin complexes are present in synovial fluids of rheumatoid arthritis patients

**Figure 4:**
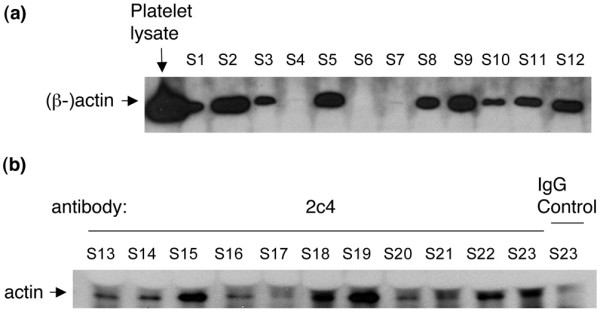
Actin and gelsolin (GSN)-actin complexes in synovial fluid (SF). **(a) **Representative immunoblot showing actin in SFs of rheumatoid arthritis (RA) patients. The beta isoform of actin is present to various degrees in SF from RA patients. **(b) **Representative immunoblot showing results from immunoprecipitation of GSN-actin complexes using the 2c4 anti-GSN antibody. SF number 23 (S23) was incubated with 2c4 or control IgG. Note that, when the band densities for S23 incubated with 2c4 versus control IgG are compared, the 2c4 antibody is seen to pull down more actin than the control IgG, indicating that actin is bound to GSN in SF.

Various amounts of actin were detected in SFs of 77 of the 81 RA patients and in plasmas of 19 of the 78 patients by immunoblotting (Figure 4a). In addition, very low concentrations of actin were detected in 2 of the 62 plasmas from healthy controls (data not shown). Concentrations of actin in patient samples were determined using purified human actin as a standard and ranged between 1 and 288 mg/L in SF and between 1 and 268 mg/L in plasma. Immunoprecipitation using the 2c4 anti-GSN antibody revealed the presence of GSN-actin complexes in SFs of RA patients (Figure 4b).

## Discussion

This is the first study to show that pGSN levels are reduced in plasma of patients with RA. The decrease is inversely related to the intensity of systemic inflammation, determined by an inverse correlation with levels of acute-phase protein (CRP). In contrast, local inflammation measured by IL-6 levels in SF has no direct correlation with pGSN levels. The finding that circulating pGSN levels decrease during chronic joint inflammation is consistent with observations of acute inflammatory disease states, such as sepsis and acute respiratory distress syndrome, in which a drop in pGSN concentration precedes more severe injuries [5-14] and the beneficial action of pGSN during re-administration in traumatized animals suggests that it has a protective role in inflammation [12,14,20]. The mechanism behind this demonstrated protective effect during acute inflammation is unclear but may reside in its binding and inactivation of inflammatory mediators) [21-26].

**Figure 5:**
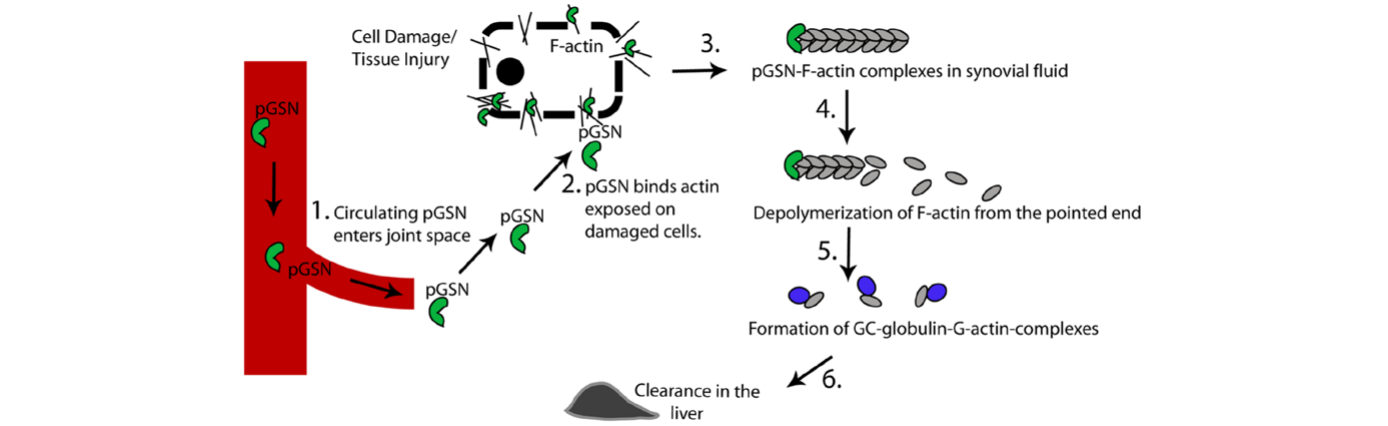
Proposed model of plasma gelsolin (pGSN) function in clearing actin from synovial joints of rheumatoid arthritis patients with acute joint effusion. (1) pGSN and other proteins from blood enter the inflamed synovial joint space freely due to increased permeability. (2) Actin is exposed to the extracellular environment of the joint because of tissue cell damage. pGSN gets soaked up in the exposed actin networks because of its high affinity for actin. (3) Actin filaments (F-actin) are severed by pGSN and GSN-F-actin complexes released to the synovial fluid (SF). (4) pGSN caps the barbed ends of the filaments, preventing polymerization from the fast-growing ends. Due to the presence of Gc-globulin [43], the pool of free actin monomers in SF is likely to be low, favoring depolymerization from the pointed (slow-growing) ends of the actin filaments. (5) Actin monomers released from the filaments are bound by Gc-globulin, which (6) efficiently clears it in the liver [18]. G-actin, monomeric actin.

pGSN was present in SFs of RA patients at a concentration of approximately 95 mg/L. Plasma origin is supported by immunoblotting and a correlation between pGSN levels in plasma and SF in the matched pair of samples. Mechanistically, pGSN may be consumed locally through trapping at the joint space or other affected organs in order to maintain a steady-state concentration at the site of inflammation. pGSN has the potential to interact with several macromolecules present at sites of inflammatory injury. In addition to its high affinity for actin, a prime candidate for its localization at an inflamed site containing damaged cells, pGSN binds to fibronectin [37] and fibrin [38,39], which are present at higher-than-normal concentrations in the inflamed joint space during RA [40-42]. We detected actin in 77 of the 81 SF samples and the presence of GSN-actin complexes suggests that one function of pGSN in the inflamed joint space is to sever F-actin exposed on damaged cells during tissue injury. Hypothetically, pGSN from blood would get trapped in the exposed actin networks, leading to a decrease in circulating pGSN. A fraction of the actin-bound pGSN would be expected to come loose from the network during the severing process and be detected as free-floating pGSN-actin complexes in SF. Such complexes would be interpreted as pGSN in the nucleation assay and contribute to the total pGSN concentration measured in the patient sample. pGSN stuck in the damaged tissue would not be detected. Gc-globulin, another component of the extracellular actin-scavenging system, has also been shown to be present in SF [43], suggesting a system for extracellular actin removal in the inflamed joint. Figure 5 describes this hypothesis of events. Since pGSN has previously been shown to bind to various inflammatory mediators such as platelet-activating factor, lysophosphatidic acid, and endotoxin, some of which are present at higher-than-normal levels in the inflamed synovial joint of RA patients [44], further studies should be conducted to understand how this local consumption due to compartmental actin binding affects cell activation induced by these mediators. While distribution into the SF and/or local trapping is likely to contribute to the decrease in circulating pGSN, pGSN may also be bound to a secondary yet unidentified plasma factor (for example, CRP) that may cause it to remain undetected by our functional assay.

Furthermore, MMPs, considered the major enzymes responsible for the extracellular matrix degradation, cleave pGSN *in vitro *[33,35] and are elevated during inflammation. It is possible that pGSN is cleaved by MMPs *in vivo *during RA and that this cleavage contributes to lower levels being detected. This is further supported by observations of MMP-mediated cleavage of 68-kDa secreted peptides from mutant (D187Y/N) pGSN [45,46]. MMP-induced pGSN cleavage would most likely be interpreted as a decrease in pGSN concentration in our functional assay since the nucleating activity of pGSN cut in half is weaker than for the full-length protein [47-49]. Although we did not detect proteolytic pGSN fragments by immunoblotting, it is possible that they are further degraded or rapidly cleared from the circulation.

## Conclusion

We have documented a decrease in levels of circulating pGSN in RA, the first example of such depletion during chronic inflammation. pGSN levels are even lower intra-articularly in affected joints. Although the cause of the decrease is unclear and enzymatic degradation or decreased production cannot be definitively ruled out, the capacity of pGSN to bind several factors present in the inflamed joint space and the lack of detection of proteolyic fragments suggest a local consumption. The proportion of pGSN levels in SF versus plasma, in combination with the observations that actin is exposed to the extracellular environment during RA and that GSN-actin complexes are present in SF, supports this hypothesis. However, the lack of a direct relation between the degree of local inflammation as measured by IL-6 and pGSN levels suggests that the regulation of pGSN in RA is complex. Further studies on the involvement of pGSN in RA might help in understanding the pathogenesis and possibly aid in diagnosis and future treatments.

## Abbreviations

cGSN: cytoplasmic gelsolin; cMMP-3: catalytic domain of matrix metalloproteinase-3; CRP: C-reactive protein; DMARD: disease-modifying anti-rheumatic drug; F-actin: filamentous actin; GSN: gelsolin; HRP: horseradish peroxidase; IL-6: interleukin-6; MMP: matrix metalloproteinase; MTX: methotrexate; pGSN: plasma gelsolin; PVDF: polyvinylidene difluoride; RA: rheumatoid arthritis; rhpGSN: recombinant human plasma gelsolin; SB: sample buffer; SF: synovial fluid.

## Competing interests

The authors' institutions, Brigham & Women's Hospital and The University of Gothenburg, have filed a patent application on which TMO, TPS, and AT are designated as inventors. The application concerns the diagnostic and therapeutic utility of pGSN measurements and administration, respectively, in inflammatory disorders. This application has been licensed to Critical Biologics Corporation (Cambridge, MA, USA), of which TPS is a founding scientist. He has stock and stock options in and receives fees from this company. The other authors declare that they have no competing interests.

## Authors' contributions

TMO performed laboratory experiments, designed experiments, and drafted the manuscript. MV participated in the statistical evaluation of data and laboratory experiments. TPS aided with important discussions, suggestions for experiments, and critical revisions of the manuscript. AT initiated, designed, and coordinated the study. MB collected and clinically characterized patient and control material, performed statistical evaluation of data, and helped to draft the manuscript. All authors read and approved the final manuscript.
